# Supplementary material for: Direct Imaging of Radiation-Sensitive Organic Polymer-Based Nanocrystals at Sub-Ångström Resolution
Source: Nanomaterials (Basel). 2024 May 17;14(10):872. doi: 10.3390/nano14100872 (PMC11539949; doi:10.3390/nano14100872)

checkCIF/PLATON report

You have not supplied any structure factors. As a result the full set of tests cannot be run.

THIS REPORT IS FOR GUIDANCE ONLY. IF USED AS PART OF A REVIEW PROCEDURE FOR PUBLICATION, IT SHOULD NOT REPLACE THE EXPERTISE OF AN EXPERIENCED CRYSTALLOGRAPHIC REFEREE.

No syntax errors found. CIF dictionary Interpreting this report

Datablock: X4ESxT-30Jul183541_a_tw6_sx

Bond precision: C-C = 0.0119 A Wavelength=0.70000

Cell:	a=6.9300(14)	b=9.3400(19)	c=19.320(4)
	alpha=86.74(3)	beta=82.59(3)	gamma=86.90(3)
Temperature:	100 K		

	Calculated	Reported
Volume	1236.7(4)	1236.7(4)
Space group	P -1	P -1
Hall group	-P 1	-P 1
Moiety formula	C8 H10 N4 O2, 2(C7 H6 F N O2), C2 H4 O	C8 H10 N4 O2, 2(C7 H6 F N O2), C2 H4 O
Sum formula	C24 H26 F2 N6 O7	C24 H26 F2 N6 O7
Mr	548.51	548.51
Dx,g cm-3	1.473	1.473
Z	2	2
Mu (mm-1)	0.114	0.114
F000	572.0	572.0
F000'	572.31	
h,k,lmax	8,11,22	8,11,22
Nref	4369	4196
Tmin,Tmax	0.993,0.998	
Tmin'	0.989	

Correction method= Not given

Data completeness= 0.960 Theta(max)= 24.624

R(reflections)= 0.1374(2984) wR2(reflections)= 0.3911(4196)

S = 1.075 Npar= 360

The following ALERTS were generated. Each ALERT has the format
test-name_ALERT_alert-type_alert-level.
Click on the hyperlinks for more details of the test.

Alert level B

```
PLAT084_ALERT_3_B High wR2 Value (i.e. > 0.25) ...................       0.39 Report
PLAT340_ALERT_3_B Low Bond Precision on  C-C Bonds ...............    0.01194 Ang.
```

Alert level C

```
PLAT029_ALERT_3_C _diffrn_measured_fraction_theta_full value Low .      0.960 Why?
PLAT052_ALERT_1_C Info on Absorption Correction Method   Not Given     Please Do !
PLAT082_ALERT_2_C High R1 Value ..................................       0.14 Report
```

Alert level G

```
ABSMU01_ALERT_1_G  Calculation of _exptl_absorpt_correction_mu
                not performed for this radiation type.
PLAT002_ALERT_2_G Number of Distance or Angle Restraints on AtSite          1 Note
PLAT004_ALERT_5_G Polymeric Structure Found with Maximum Dimension          1 Info
PLAT007_ALERT_5_G Number of Unrefined Donor-H Atoms ..............          6 Report
PLAT083_ALERT_2_G SHELXL Second Parameter in WGHT  Unusually Large       5.66 Why ?
PLAT092_ALERT_4_G Check: Wavelength Given is not Cu,Ga,Mo,Ag,In Ka    0.70000 Ang.
PLAT154_ALERT_1_G The s.u.'s on the Cell Angles are Equal ..(Note)       0.03 Degree
PLAT172_ALERT_4_G The CIF-Embedded .res File Contains DFIX Records          1 Report
PLAT180_ALERT_4_G Check Cell Rounding: # of Values Ending with 0 =          4 Note
PLAT300_ALERT_4_G Atom Site Occupancy of H6A       Constrained at         0.5 Check
PLAT300_ALERT_4_G Atom Site Occupancy of H6B       Constrained at         0.5 Check
PLAT300_ALERT_4_G Atom Site Occupancy of H6C       Constrained at         0.5 Check
PLAT300_ALERT_4_G Atom Site Occupancy of H6D       Constrained at         0.5 Check
PLAT300_ALERT_4_G Atom Site Occupancy of H6E       Constrained at         0.5 Check
PLAT300_ALERT_4_G Atom Site Occupancy of H6F       Constrained at         0.5 Check
PLAT300_ALERT_4_G Atom Site Occupancy of H7A       Constrained at         0.5 Check
PLAT300_ALERT_4_G Atom Site Occupancy of H7B       Constrained at         0.5 Check
PLAT300_ALERT_4_G Atom Site Occupancy of H7C       Constrained at         0.5 Check
PLAT300_ALERT_4_G Atom Site Occupancy of H7D       Constrained at         0.5 Check
PLAT300_ALERT_4_G Atom Site Occupancy of H7E       Constrained at         0.5 Check
PLAT300_ALERT_4_G Atom Site Occupancy of H7F       Constrained at         0.5 Check
PLAT300_ALERT_4_G Atom Site Occupancy of H8A       Constrained at         0.5 Check
PLAT300_ALERT_4_G Atom Site Occupancy of H8B       Constrained at         0.5 Check
PLAT300_ALERT_4_G Atom Site Occupancy of H8C       Constrained at         0.5 Check
PLAT300_ALERT_4_G Atom Site Occupancy of H8D       Constrained at         0.5 Check
PLAT300_ALERT_4_G Atom Site Occupancy of H8E       Constrained at         0.5 Check
PLAT300_ALERT_4_G Atom Site Occupancy of H8F       Constrained at         0.5 Check
PLAT860_ALERT_3_G Number of Least-Squares Restraints .............          1 Note
PLAT870_ALERT_4_G ALERTS Related to Twinning Effects Suppressed ..          ! Info
PLAT883_ALERT_1_G No Info/Value for _atom_sites_solution_primary .     Please Do !
PLAT933_ALERT_2_G Number of HKL-OMIT Records in Embedded .res File         56 Note
PLAT941_ALERT_3_G Average HKL Measurement Multiplicity ...........        1.0 Low
PLAT965_ALERT_2_G The SHELXL WEIGHT Optimisation has not Converged     Please Check
```

0 **ALERT level A** = Most likely a serious problem - resolve or explain
2 **ALERT level B** = A potentially serious problem, consider carefully

```
 3 ALERT level C = Check. Ensure it is not caused by an omission or oversight
33 ALERT level G = General information/check it is not something unexpected

 4 ALERT type 1 CIF construction/syntax error, inconsistent or missing data
 5 ALERT type 2 Indicator that the structure model may be wrong or deficient
 5 ALERT type 3 Indicator that the structure quality may be low
22 ALERT type 4 Improvement, methodology, query or suggestion
 2 ALERT type 5 Informative message, check
```

It is advisable to attempt to resolve as many as possible of the alerts in all categories. Often the minor alerts point to easily fixed oversights, errors and omissions in your CIF or refinement strategy, so attention to these fine details can be worthwhile. In order to resolve some of the more serious problems it may be necessary to carry out additional measurements or structure refinements. However, the purpose of your study may justify the reported deviations and the more serious of these should normally be commented upon in the discussion or experimental section of a paper or in the "special_details" fields of the CIF. checkCIF was carefully designed to identify outliers and unusual parameters, but every test has its limitations and alerts that are not important in a particular case may appear. Conversely, the absence of alerts does not guarantee there are no aspects of the results needing attention. It is up to the individual to critically assess their own results and, if necessary, seek expert advice.

Publication of your CIF in IUCr journals

A basic structural check has been run on your CIF. These basic checks will be run on all CIFs submitted for publication in IUCr journals (*Acta Crystallographica*, *Journal of Applied Crystallography*, *Journal of Synchrotron Radiation*); however, if you intend to submit to *Acta Crystallographica Section C* or *E* or *IUCrData*, you should make sure that full publication checks are run on the final version of your CIF prior to submission.

Publication of your CIF in other journals

Please refer to the *Notes for Authors* of the relevant journal for any special instructions relating to CIF submission.

PLATON version of 28/11/2022; check.def file version of 28/11/2022

Datablock X4ESxT-30Jul183541_a_tw6_sx - ellipsoid plot

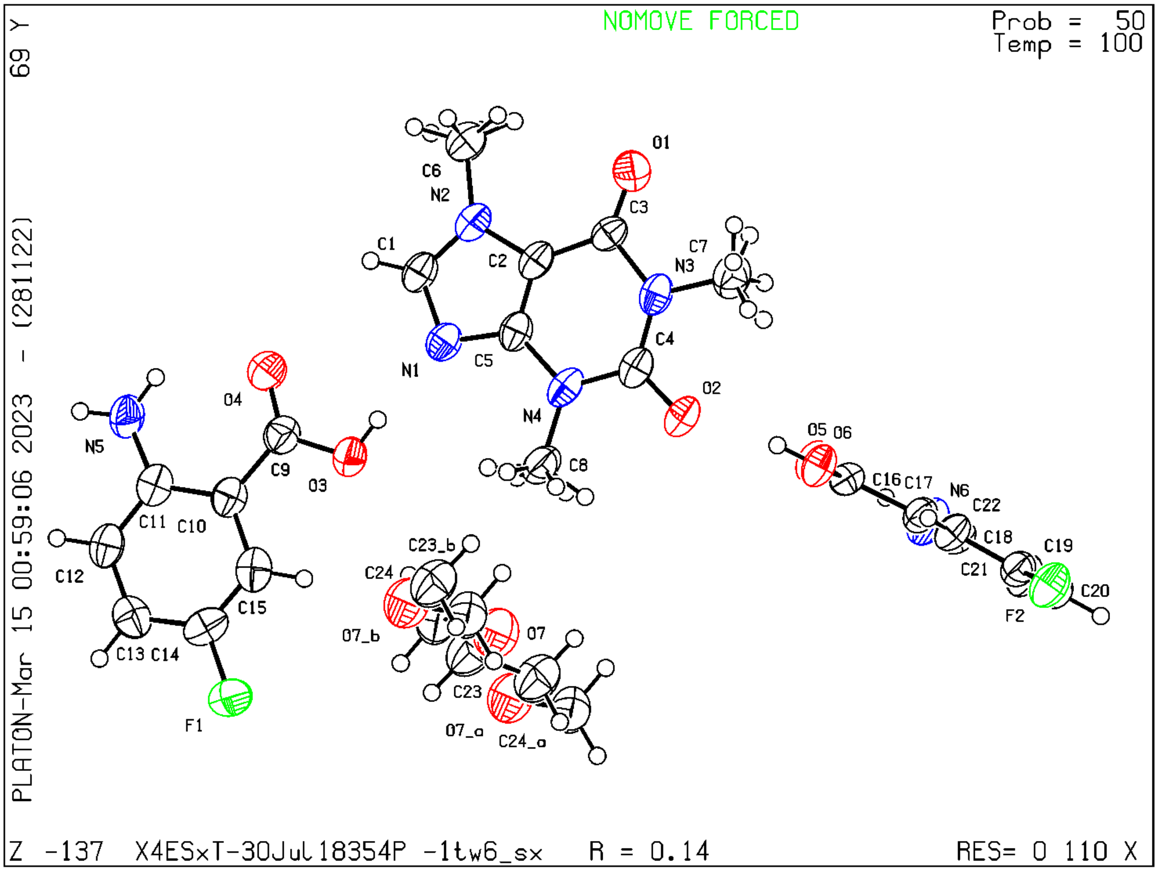

Supplement: Supplementary file 1 [file nanomaterials-14-00872-s001.zip › SM archive/checkcif_CAPeg.pdf]
