# Supplementary material for: Direct Imaging of Radiation-Sensitive Organic Polymer-Based Nanocrystals at Sub-Ångström Resolution
Source: Nanomaterials (Basel). 2024 May 17;14(10):872. doi: 10.3390/nano14100872 (PMC11539949; doi:10.3390/nano14100872)
Supplement: Supplementary file 1 [file nanomaterials-14-00872-s001.zip › SM archive/nanomaterials-2995111-supplementary.pdf]

Supplementary Information for

Direct Imaging of Radiation Sensitive Organic Polymer-Based Nanocrystals at Sub-Ångström Resolution

E. Carlino[1]*, A. Taurino[2], D. Hasa[3], D.-K. Bučar[4], M. Polentarutti[5], L. E. Chinchilla[6], J. J. Calvino Gamez[6]
*Corresponding author: elvio.carlino@cnr.it

1. Mechanochemical synthesis of polymer-based cocrystals

Anhydrous caffeine (caf) (99%), 5-fluoroanthranilic acid (5Fana) (97%), poly(ethylene glycol) dimethyl ether 1000 (PEG-DME) (M_n=1050 g mol^{-1}), poly(ethylene glycol)-poly(propylene glycol) copolymer (PEG-PPG) (M_n=8400 g mol^{-1}) were purchased from Sigma Aldrich and used as received. In a typical mechanochemical experiment, a physical mixture (m = 250 mg) composed of caffeine, 5Fana and either PEG-DME or PEG-PPG was added to a 15 ml screw stainless steel milling jar with two 7 mm steel milling balls, and subsequently milled for 120 min at 25 Hz.

2. Preparation of a single crystal of CAPeg

Single crystals of mechanochemically prepared CAPeg cocrystal batch were prepared through melt crystallization in DSC pan. Approximately 1.5 mg of solid was placed in a DSC pan, and heated at 140°C at a heating rate of 10°C/min, followed by an isothermal stage of 30 min. The melt was subsequently cooled down to 0°C with a cooling rate of 0.5°C/min. The obtained crystals were analyses using PXRD before being subjected to single crystal X-ray diffraction analyses (see Fig. **S1**, **S2**, and **S3**) and Differential scanning calorimetric measurements (Fig. **S4**).

3. Single crystal X-ray diffraction studies

The X-ray diffraction measurements for CAPeg were pursued at the XRD1[1] beamline Elettra Sincrotrone Trieste (Italy). The XRD1 beamline is equipped with a *Huber* kappa goniometer and a *Dectris Pilatus* 2M detector. A photon energy of 17.712 keV (λ = 0.7000 Å) was selected for the data collection. The diffraction data were integrated with the XDS program[2] using default parameters. The same software was used for an empirical absorption correction using spherical harmonics on symmetry-equivalent and redundant data, and the correction for Lorentz and polarization effects. The space group determination was accomplished using the Pointless programme from the CCP4 software suite.

The crystal structure was solved with the *SHELXT* programme,[3] used within the *Olex2* software suite,[4] and refined by least squares based on F^2 with the *SHELXL* programme[5] using the *ShelXle* graphical user interface.[6] All non-hydrogen atoms were refined anisotropically by the full-matrix least-squares method. Hydrogen atoms associated with carbon, nitrogen and oxygen atoms were refined isotropically [$U_{iso}(H_{C,N}) = 1.2 \cdot U_{eq}(C,N)$, $U_{iso}(H_O) = 1.5 \cdot U_{eq}(O)$] in geometrically constrained positions. The crystallographic and refinement parameters of CAPeg are given in Table **S1**. The asymmetric units of the crystal structures of CAPeg are shown in Figure **S5**.

4. TEM sample preparation for HoloTEM

There are two main requirements for TEM sample preparation that must be fulfilled for atomic resolution HoloTEM experiments on radiation sensitive nanoparticles:

1) The density of particles on TEM grid must be low enough to avoid particles overlapping for a true single particle imaging.
2) The eventual dispersion of particles in a liquid, according to well established procedure for TEM grids preparation,[7] should not modify the pristine structure of the material.

Here we studied polymeric cocrystals containing caffein and anthranilic acid, which are both highly soluble in many solvents commonly used for particle dispersions necessary for particle deposition on TEM supports, and hence dispersion in liquid was avoided. Consequently, pristine powders were finely grinded in agate mortar and transferred on a TEM Cu grid, covered by thin C foil, by using a needle. This procedure and a certain skill enable to deposit the right density of

pristine nanoparticles on the grid. The sample so obtained is not suitable to be immediately inserted into the TEM for the experiments, as we observed a very high rate of hydrocarbon contamination that jeopardizes HoloTEM experiments. The sample needs hence to be left under vacuum to degas for at least 8 hours. In our experiments we noted that a small residual rate of hydrocarbon contamination,[8] whose thickness does not interfere too much with the HoloTEM experiments, is helpful to reduce the radiation damage in the specimen. In fact, we observed that sample without the formation of a small protective layer of contaminants are much more sensitive to radiation damage. This is not so surprising, as reported by Egerton, as coating both sides of a TEM specimen with carbon (or a metal) has been shown to have a protective effect, reducing mass loss.[9] A possible explanation is that the coating acts as a diffusion barrier, reducing the escape rate for light gaseous elements. Researchers studying the composition of interfaces (using a highly focused electron probe) learned to pre-irradiate the area of interest in an older TEM, producing a thin contamination layer on each surface, which inhibits the loss of light elements. More surprisingly, surface coating is also found to reduce the loss of crystallinity. One proposed explanation is that return to the original molecular state (healing of the broken bond) is more likely if the escape of volatile elements is prevented.[10] Increasing the thickness of a specimen should also reduce the out-diffusion rate and indeed the dose required to destroy crystallinity has been found to increase with increasing thickness.[11] For inorganic materials, Strane et al.[12] have suggested that coating reduces the rate of desorption-induced electronic transitions (DIET). Coating might also reduce beam-induced temperature rise or electrostatic charging,[13] besides acting as sputtering barrier.

5. Experimental HoloTEM

HoloTEM atomic resolution experiments on different kind of polymeric cocrystals were performed using a double spherical aberration corrected FEI Titan 60-300 Themis microscope operated at 200 kV using a spot size 3, and 150 μm C2 aperture. The aberration corrector was tuned prior to inserting the sample and the correction of the spherical aberration of the objective lens allows a spatial resolution of 0.08 nm in HRTEM imaging. The beam intensity was adjusted to the maximum diameter within parallel illumination conditions for a 3-condenser lens system and gave the desired dose for the dataset. The microscope is equipped with a Direct Electron Detection Gatan K3 IS system model 1026, which contains a complementary metal-oxide semiconductor (CMOS) detector, and high-speed electronics able to recognize, locate and count each electron event at about 1500 frames per second. Each dataset was recorded in counting mode, which enables to operate the beam at lowest level of irradiation rate, between 4 - 40 e^-/pixel/s. In addition to imaging experiments, chemical analysis was carried out using the X-ray Energy Dispersed Spectrometer (EDXS) arranged by 4 windowless silicon drift detectors, providing a total sensor area of 120 mm^2 and a solid collection angle of 0.7sr. All the experiments were performed at room temperature. HRTEM images and diffraction patterns were simulated in both Bloch's waves[14] and Multislice[15] approaches by Java Electron Microscopy Simulation (JEMS) program.[16]

6. Additional on HoloTEM experiments, HRTEM image Simulations and EDXS experimental results.

6.1 Use of multiple HRTEM image acquisition to check the possible specimen damage.

The occurrence of particle damage when present is usually evident inspecting the sum HRTEM image and the relevant diffractogram as acquired. In this case the relevant data are usually immediately discarded to avoid waste of hard disk memory. Possible subtle specimen damage is monitored offline during the data analysis by using the multiple HRTEM image

acquisition allowed by the high sensitivity and high acquisition frame rate of the direct detection Gatan K3 camera. Each HRTEM image has a typical exposure time of ~0.01 s and the electron density rate is about ~100 $e^{-}/s \cdot Å^{2}$, resulting in a density of electrons for each HRTEM image from 1 to 3 $e^{-}/Å^{2}$. A total of 100-200 HRTEM images for each area of interest is hence acquired. Fig. **S6** is an experimental example of the procedure to detect subtle particle damage. In this case a total of 100 HRTEM images was acquired with a total exposure time of 2s. Each HRTEM image was exposed to a total density of electrons of 2.4 $e^{-}/Å^{2}$. Fig. **S6 a)** is the first HRTEM image of the series shown together with the relevant diffractogram, **b)** is the tenth image of the series shown together with the relevant diffractogram, **c)** is the fiftieth image of the series shown with the relevant diffractogram, **d**) is the hundredth image of the series with the relevant diffractogram and finally **e)** is the HRTEM sum of all the 100 images. The image features within each single HRTEM image have a small contrast due to the little number of electrons used, nevertheless the signal is enough to distinguish the lattice fringes and to compare the images. Possible crystalline features modification can also be evaluated comparing each diffractogram with the previous one. A numerical evaluation can also be performed by using the statistical features of the Digital Micrograph Suite 3.43™.[17] For reader convenience the original .DM4 data for the series of Fig. **S6** can be found in the supplementary materials. The presence of possible particle damage can also be monitored by using the routine within Digital Micrograph Suite 3.43 available to check eventual drift in the HRTEM series, the routine cross correlate each image with the previous one to detect eventual changes.

6.2 High magnification image, hologram and diffractogram of the particle in Fig. 1

Fig. **S7** details, at high magnification for reader convenience, both the HRTEM image in the Scheme 1 and, as inset, the relevant raw hologram. While in the hologram the particle is sharply visible with a total irradiation of 1.2$e^{-}/Å^{2}$, to have enough contrast in the HRTEM image it was necessary summing 98 frames, each of them exposed to a density of current of 1.2$e^{-}/Å^{2}$, for a total exposure time of 1.5 s. This marks how, on specimen made by small nanoparticles of pristine organic matter, high magnification multibeam imaging is practically unusable for the specimen survey.

6.3 Conditions for the simulation of HRTEM image and diffraction pattern in Fig. 2.

The experimental diffractogram has been simulated by full dynamical calculations in the multislice framework using JEMS.[16] As input data for the calculation, the structural file derived by the synchrotron XRD measurements was used (see table **S1**). Good match has been found for $[3,6,\bar{4}]$ zone axis and a specimen thickness of 2.3 nm. Hence, the same zone axis and thickness have been used to calculate the HRTEM lattice fringes contrast, which gave a good match for an objective lens defocus = 10.1 nm. The other experimental parameters used as input in the calculations are: spherical aberration coefficient Cs=3.26 μm, twofold astigmatism = 1.9 nm, threefold astigmatism = 63.3 nm, second order axial coma = 24.9 nm.

6.4 Conditions for the simulation of HRTEM image and diffraction pattern in Fig. 3.

Good match between experimental diffractogram and simulated diffraction pattern has been found for [10, 3, 2] zone axis, as shown in Fig. **S8** where the experimental and simulated pattern are superimposed to evidence their agreement. The imaged region in Fig.3c contains areas with different thicknesses: in particular, the area in the upper-left part was successfully simulated for a thickness of 27nm whereas the region in the lower-central part of the image matched the simulation considering a thickness of 18nm. For both simulations the defocus used was 2.6 nm. The other experimental parameters used as input in the calculations are: spherical

aberration coefficient Cs=3.26 μm, twofold astigmatism = 1.9 nm, threefold astigmatism = 63.3 nm, second order axial coma = 24.9 nm.

6.5 Strain in the CAPeg foil.

The visibility of tensile and compressive strain in relatively large foil of CAPeg need the use of a regular grating superimposed to the experimental HRTEM image as shown in Fig. **S9**. As a guide for the eyes, a pale-blue cross-grating was superimposed to the HRTEM image to highlight the lattice deformation in polymeric CAPeg Cocrystals. The yellow spaced dashed-lines mark the set of (0, -2, 3) lattice fringes and the red dashed-lines mark the set of (1, -2, -2) lattice fringes. The arrows point some of the areas where the fringes are out from the grating due to the crystal deformation.

6.6 Radiation damage in well oriented PEG polymer particle.

Our experiments evidence the basic role of the scattering conditions of the particle on the total density of electrons that the particle can receive before evidencing a structural damage. The contrast of the particle in the hologram is function of its scattering conditions and hence particles well oriented along a zone axis can be selected for the HRTEM imaging during the live survey by the hologram. We noticed that, when the particle is not well oriented, it suffers a structural disruption for densities of electrons comparable to those expected from existing literature. On the contrary, when the particle is well oriented along a zone axis the dose that the particle can receive before evidencing a structural damage is orders of magnitude higher. We also noticed difference between different zone axes. A quantitative exhaustive study on the influence of the difference on the particle radiation robustness depending on the channeling conditions are not in the scope of this paper. Nevertheless, to highlight this dependance, which is important to understand the features of the results reported in our study, we performed some experiments on pristine nanoparticles of PEG, a polymer whose sensitivity to radiation damage is well known in literature. In Fig. **S10** we show an HRTEM series on a crystalline nanoparticle of pure PEG with its lattice well oriented for HRTEM. We show in a) a snapshot together with its diffractogram as acquired delivering a total dose of 120 $e^-/Å^2$. The snapshot in b) is acquired after that a total density of electron of 360$e^-/Å^2$ was delivered to the particle. We caution the reader that the two images were acquired using different focus conditions, which results in a sharp Fresnel's fringe visible in the first image. Nevertheless, the accurate inspection of the images and of the relevant diffractograms shows similar structural features. The third snapshot acquired after a total exposure of the particle to a density of electrons of $1.16x10^3$ $e^-/Å^2$ still evidences large areas of crystalline order despite the presence of some amorphisation at the boundary of the particle and the disappearance of some intensities in the relevant diffractogram. Crystalline areas with some further amorphisation are still visible in d) after delivering a total dose of electrons of 26.4 $x10^3$ $e^-/Å^2$. Finally, a total dose of $85.4x10^3$ $e^-/Å^2$ causes the total amorphisation of the particle except for some very small area, as evidenced also by the relevant diffractogram. From the data in Fig. **S10** it is evident how is crucial the influence of the diffraction condition on the threshold for structural damage in crystalline nanoparticles of soft-matter.

6.7 Energy dispersive X-ray spectroscopy experiments.

Reliable quantitative Energy Dispersive X-ray Spectroscopy (EDXS) on CAP and CAPeg requires dose of electrons that damage the particles, nevertheless some spectra were acquired to cross-check qualitatively the chemistry of the specimens regardless the observed disruption of the crystal structure. Fig. **S11** shows EDXS representative spectra from CAPeg, together with the HRTEM images and diffractograms of the particles collected before the EDXS spectra

acquisition. The EDXS peaks in Fig. **S11** show the presence of the expected species plus some peaks around 1 keV: the peak at 930 eV is due to spurious x-rays coming from the TEM Cu-grid whereas the peak at about 1040 eV is due to contribution of Na contaminants, present in the materials as purchased, plus eventually some spurious x-rays coming from the specimen holder.

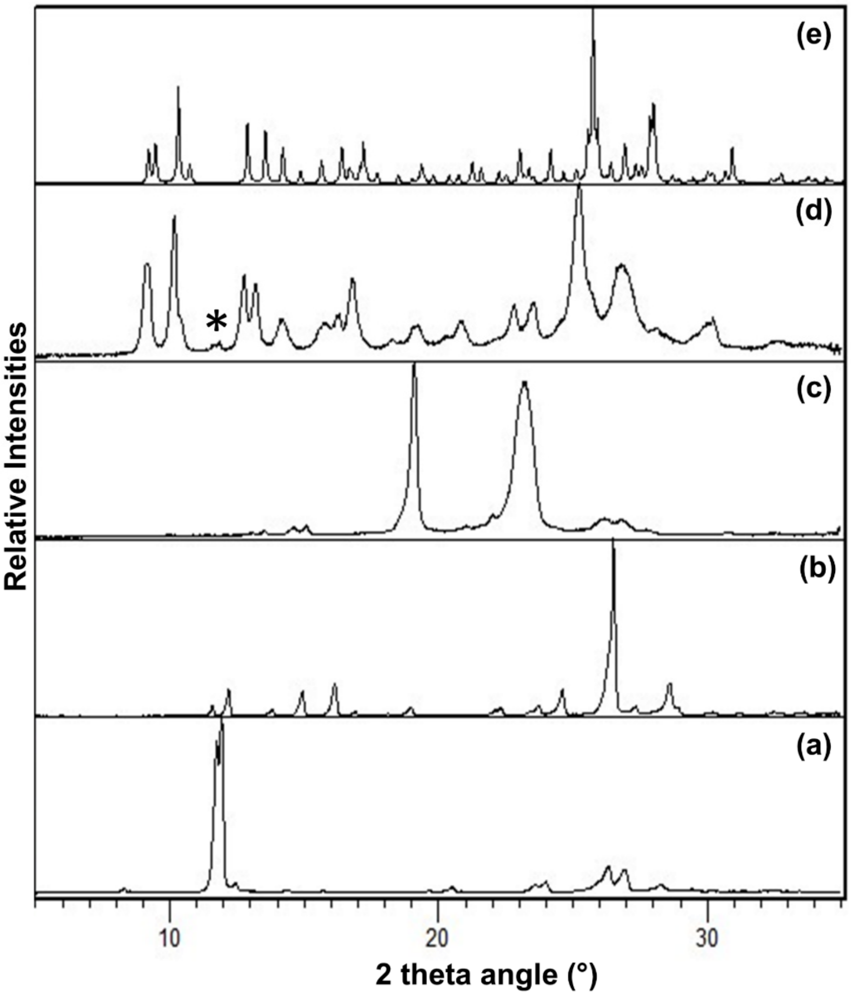

Figure S1. XRD measurements

Measured diffractograms of (a) caf, (b) 5Fana, (c) PEG-DME, (d) CAPeg cocrystal, as compared to (e) its simulated diffractogram derived from single crystal X-ray diffraction data. The diffraction peak at approximately 12° of 2-theta angle (highlighted using the * symbol) corresponds to caf impurities (the structure can be found in the Cambridge Structural Database (CSD) with the reference code: NIWFEE06).

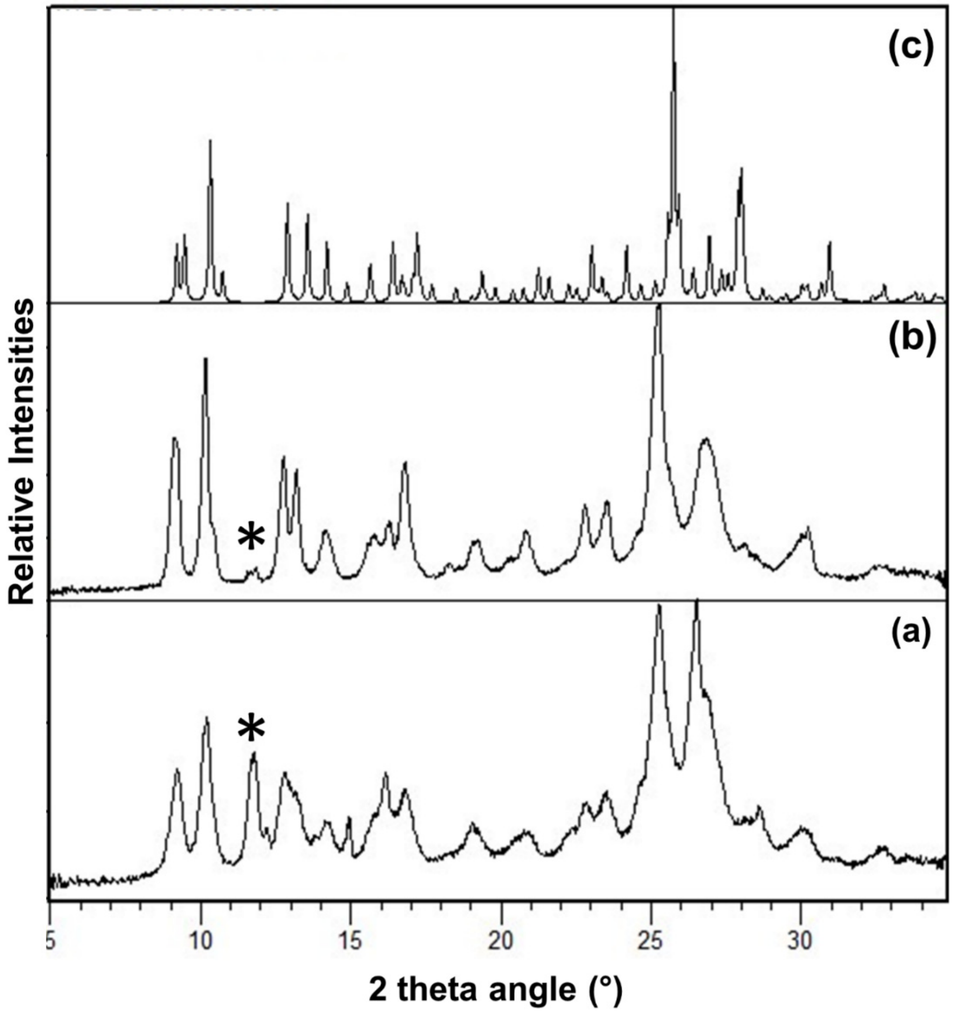

Figure S2. XRD measurements

Measured diffractograms of (a) CAPeg cocrystal, (b) CAP, as compared to (c) its simulated diffractogram derived from single crystal X-ray diffraction data. The diffraction peak at approximately 12° of 2-theta angle (highlighted using the * symbol) corresponds to caf impurities (the calculated structure can be found in the CSD with the reference code: NIWFEE06).

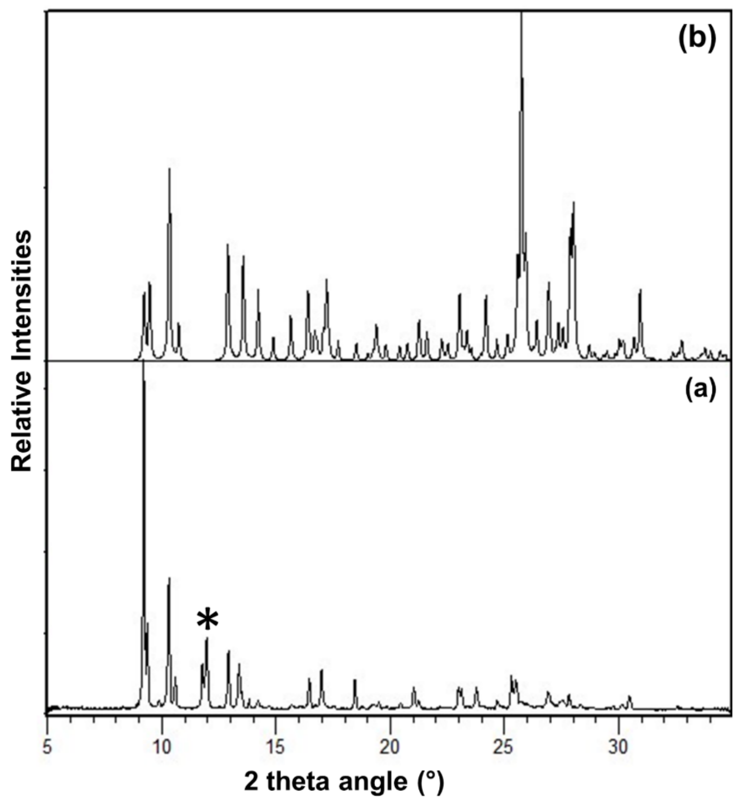

FigureS3. XRD measurements

Measured diffractogram of (a) CAPeg cocrystal recrystallized from melt, as compared to (b) its simulated diffractogram derived from single crystal X-ray diffraction data. The diffraction peak at approximately 12° of 2-theta angle (highlighted using the * symbol) corresponds to caf impurities (the calculated structure can be found in the CSD with the reference code: NIWFEE06).

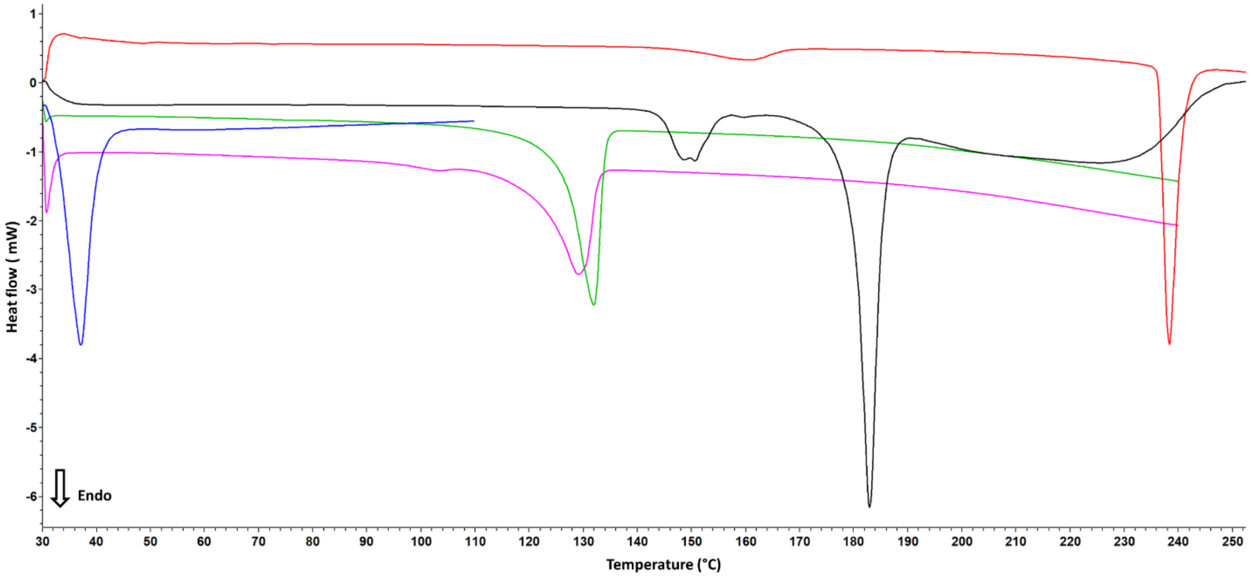

FigureS4. DSC measurements
Measured DSC thermograms of (red) caf, (black) 5Fana, (blue) PEG-DME, (green) CAPeg cocrystal and (pink) CAP cocrystal.

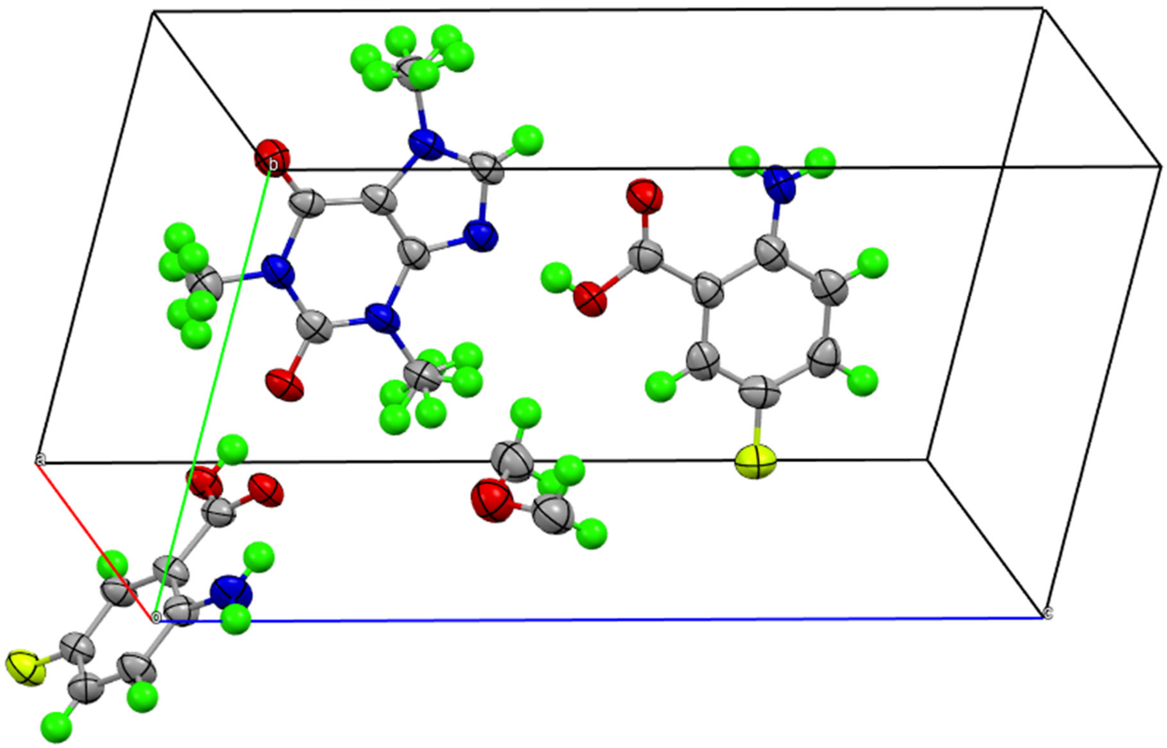

FigureS5. Structure determination
The asymmetric unit of CAPeg. The ellipsoids are drawn at a 50% probability level. Colour scheme: carbon – dark grey, hydrogen – green, nitrogen – blue, oxygen – red, fluorine – yellow.

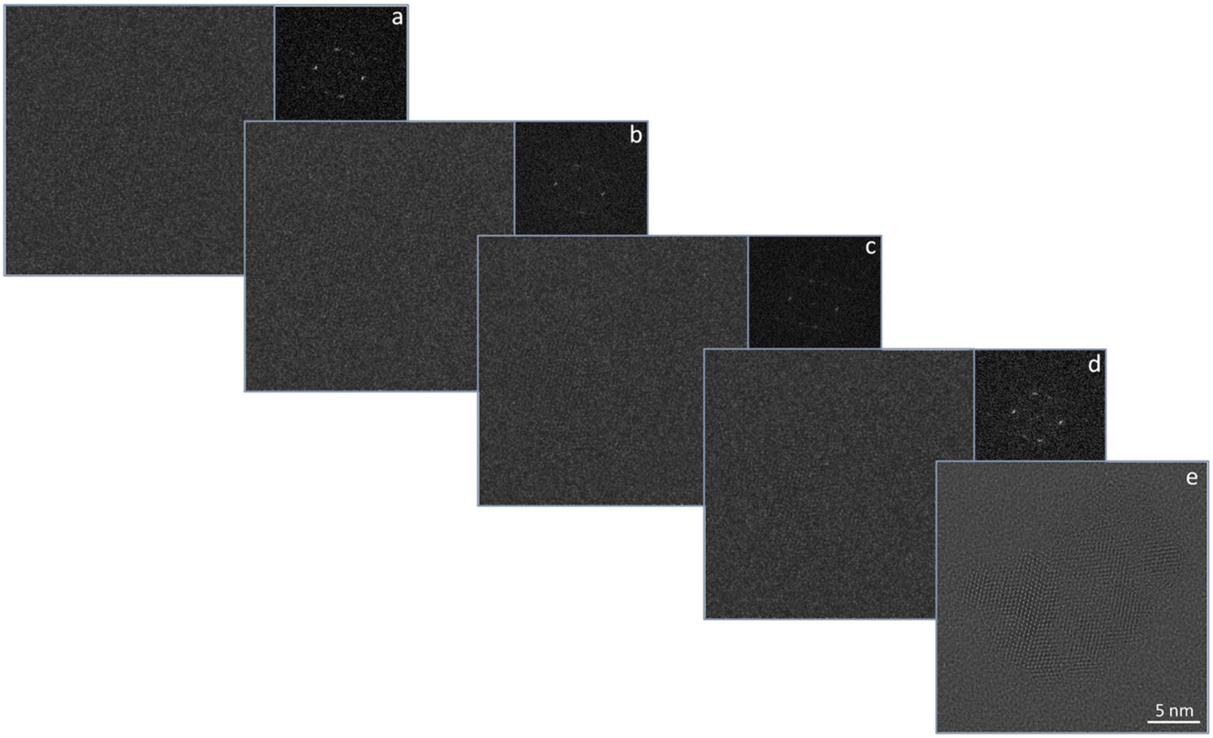

FigureS6. Example of multiple Low-dose HRTEM image series
Some of 100 low-dose HRTEM images acquired for a total exposure time of 2s. Each image received a total density of electrons of 2.4 $e^-/Å^2$. a) First image of the series and relevant diffractogram; b) Tenth image and relevant diffractogram; c) fiftieth image of the series and relevant diffractogram; d) hundredth image of the series and relevant diffractogram; e) Sum of the 100 HRTEM images. Due to the very low density of electrons the lattice fringes contrast in each image is rather low but can be seen by magnifying each image. For reader convenience the original .DM4 file of the image series was uploaded to the journal site, and can be used, within Digital Micrograph 3.43 Suite™, to measure the counts in each point of the images and of the relevant diffractograms.

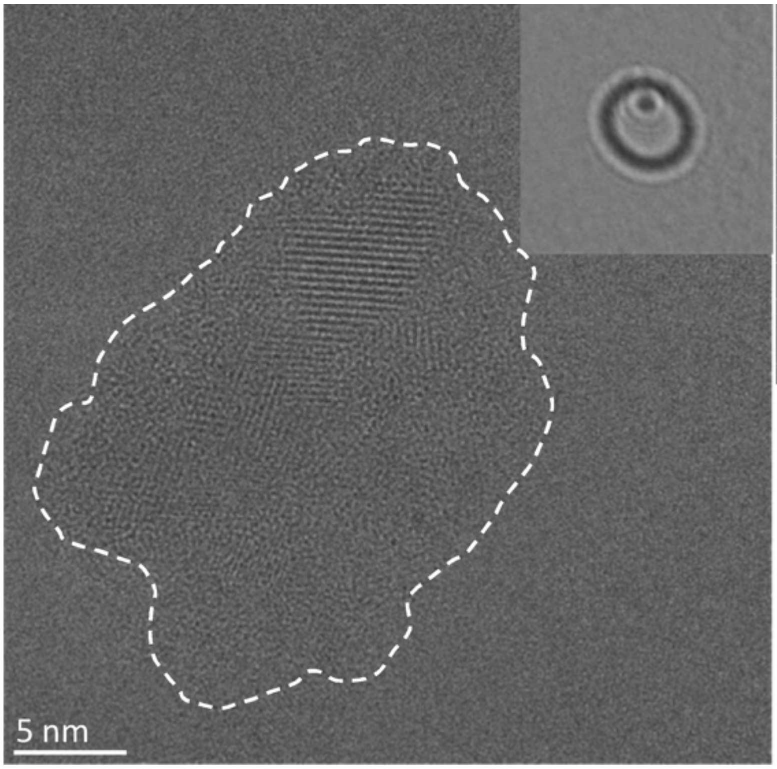

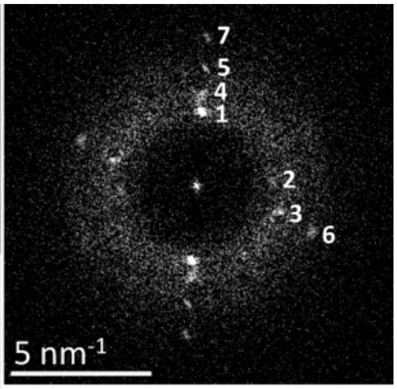

label	d_{exp} (Å)
1	3.75±0.05
2	3.54±0.05
3	3.22±0.05
4	3.02±0.05
5	2.35±0.04
6	2.28±0.04
7	1.87±0.04

FigureS7. High spatial resolution experiment on CAP multidomain nanoparticle
Left: HRTEM image of a CAP particle with the relevant hologram in the inset. The particle is the same shown in the Scheme 1 but here the HRTEM image is the sum of 98 frames each of them exposed to 2.4 $e^-/Å^2$. For reader convenience, the dashed line marks the area where the particle is. Right: diffractogram of the HRTEM image together with the lattice spacing measured.

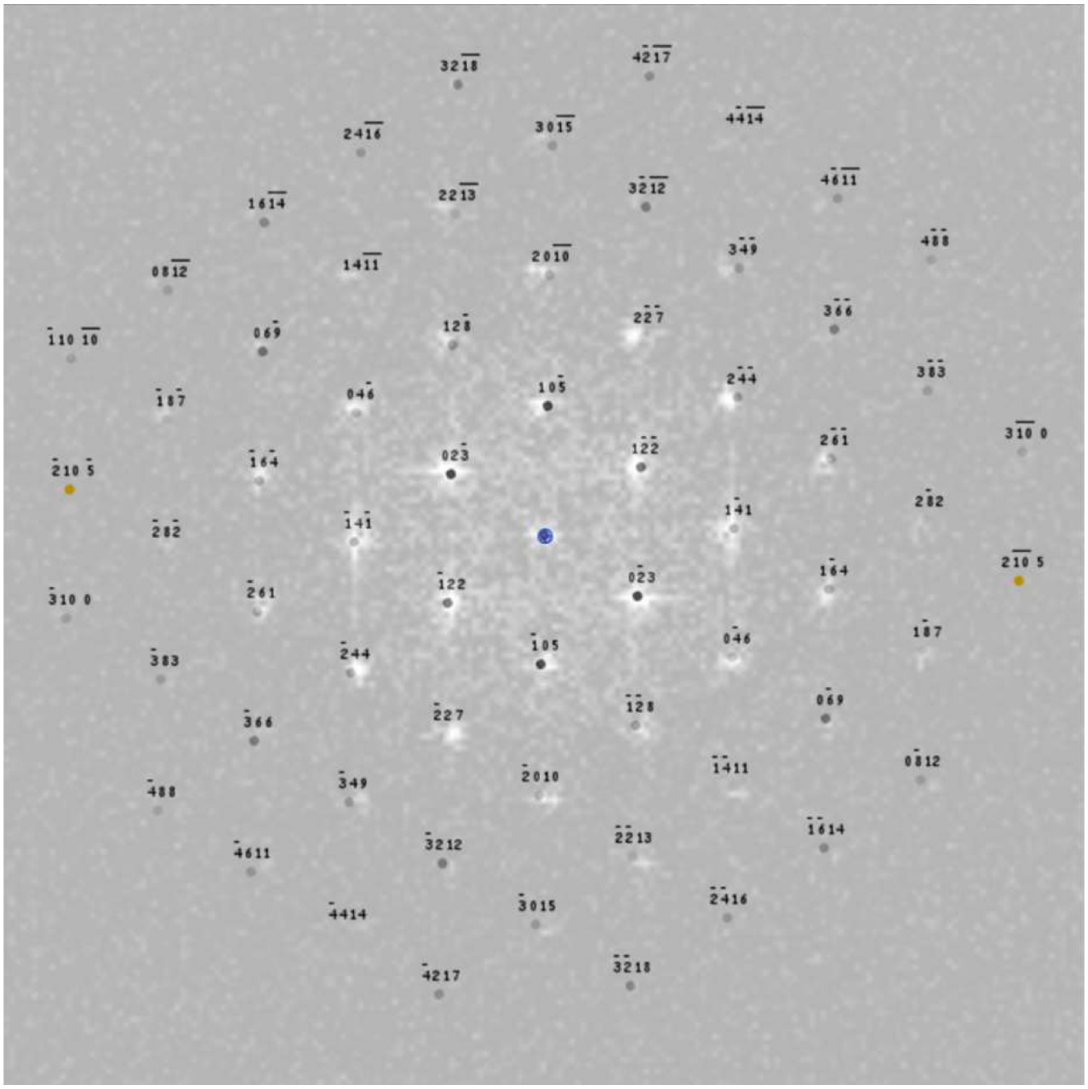

FigureS8. Simulated and experimental diffraction pattern
Simulated indexed diffraction pattern in [10, 3, 2] zone axis superimposed, for ease of comparison, to the experimental diffractogram (see also Fig. 3).

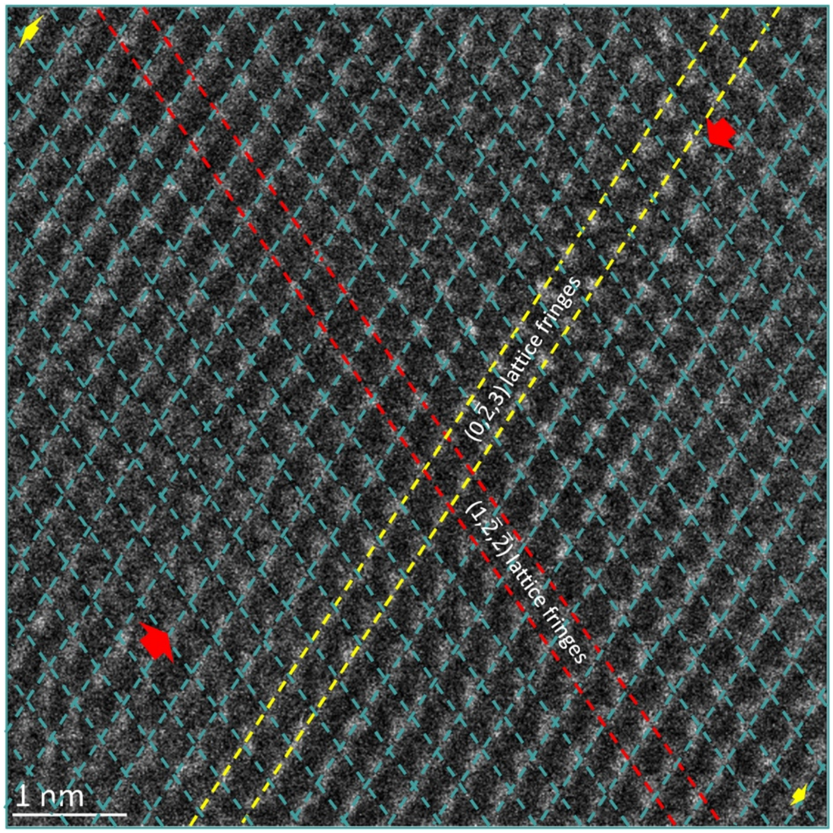

FigureS9. Strain in CAPeg foils
As a guide for the eyes, a pale-blue cross-grating was superimposed to HRTEM image to highlight the lattice deformation in polymeric CAPeg Cocrystals. The yellow spaced dashed lines mark the set of (0, -2, 3) lattice fringes and the red dashed lines mark the set of (1, -2, -2) lattice fringes. The arrows point some of the areas where the fringes are out from the grating lines due to the crystal deformation.

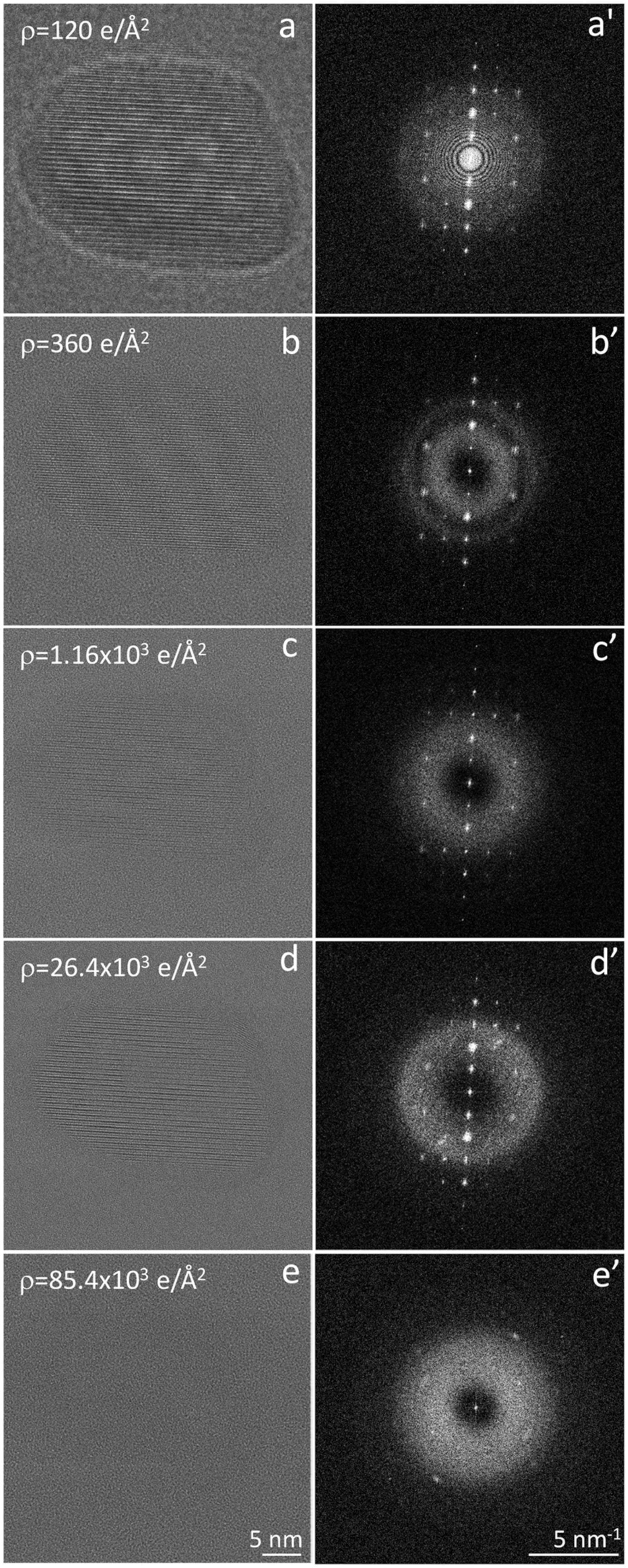

FigureS10. Radiation damage in pure PEG particle. Diffraction conditions heavily influence the particle robustness to electron irradiation

HRTEM image series, and relevant FFTs, as a function of the dose delivered to a crystalline nanoparticle PEG polymer. a) First image of the series together with its FFT. The particle has been exposed to a density of electron of $120e^-/Å^2$; b) HRTEM image and relevant FFT of the particle after its exposure to total density of electrons of $360e^-/Å^2$; c) HRTEM image and relevant FFT of the particle after its exposure to total density of electrons of $1.16x10^3e^-/Å^2$; d) HRTEM image and relevant FFT of the particle after its exposure to total density of electrons of $26.4x10^3e^-/Å^2$; e) HRTEM image and relevant FFT of the particle after its exposure to total density of electrons of $85.4x10^3e^-/Å^2$.

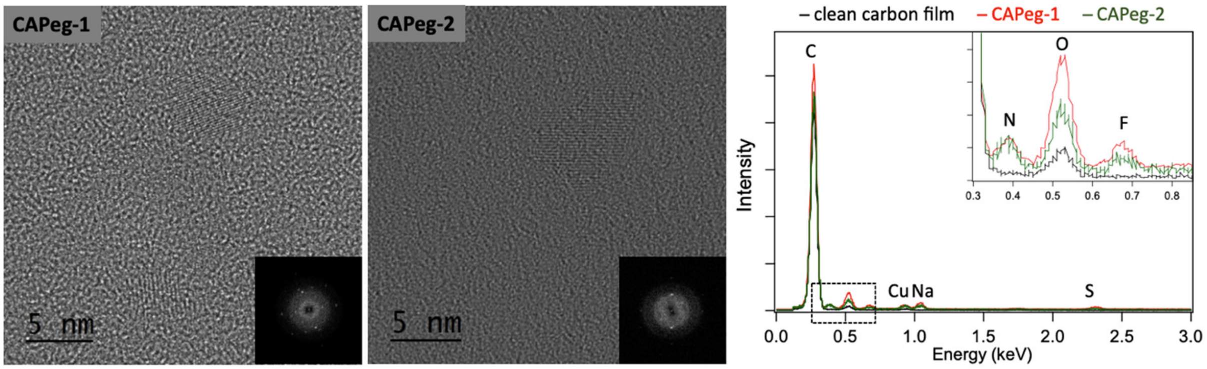

FigureS11. EDXS Spectra on CAPeg particles
Representative EDXS spectra as acquired on the particle in the HRTEM images on the left.

	CAPeg
empirical formula	$C_{24}H_{26}F_{2}N_{6}O_{7}$
M_r / g mol^{-1}	548.51
crystal system	triclinic
space group	$P\bar{1}$
a / Å	6.9300(14)
b / Å	9.3400(19)
c / Å	19.320(4)
α / °	86.74(3)
β / °	82.59(3)
γ / °	86.90(3)
V / Å^3	1236.7(4)
Z	2
ρ_{calc} / g cm^{-3}	1.473
T / K	100(2)
μ / mm^{-1}	0.114
F(000)	572
crystal size / mm^3	0.10 × 0.05 × 0.02
$\lambda_{radiation}$ / Å	0.700
2ϑ range for data collection / °	2.096 – 49.248
index ranges	$-8 \le h \le 8$<br>$-11 \le k \le 11$<br>$-22 \le l \le 22$
number of collected reflections	4196
unique reflections	4196
number of unique reflections	2984 [$I > 2\sigma(I)$]
R_{int}	0.0360
$R(F)$, $F > 2\sigma$(F)	0.1374
$wR(F^2)$, $F > 2\sigma$(F)	0.1756
$R(F)$, all data	0.3540
$wR(F^2)$, all data	0.3911
Δ_r (max., min.) e Å^{-3}	0.547/−0.501
CCDC deposition number	2248768

Table S1. Structure determination

Crystallographic and refinement parameters of CAPeg.

References:

[1] A. Lausi, M. Polentarutti, S. Onesti, J.R. Plaisier, E. Busetto, G. Bais, L. Barba, A. Cassetta, G.Campi, D. Lamba, A. Pifferi, S.C. Mande, D.D. Sarma, S.M. Sharma, G. Paolucci, Status of the crystallography beamlines at Elettra. Eur. Phys. J. Plus, 130 (2015) 43.

[2] W. Kabsch, Integration, scaling, space-group assignment and post refinement Acta Cryst. D Biol. Cryst. 66 (2010) 133–144.

[3] G. M. Sheldrick, SHELXT - Integrated space-group and crystal-structure determination. Acta Cryst. A., 64, (2015) 3–8. https://doi.org/10.1107/S2053273314026370.

[4] O.V. Dolomanov, L.J. Bourhis, R.J. Gildea, J.A.K. Howard, H. Puschmann, OLEX2: a complete structure solution, refinement and analysis program, J. Appl. Cryst. 42 (2009) 339–341.
[5] G.M. Sheldrick, Crystal structure refinement with SHELXL. Acta Cryst. C 71 (2015) 3–8. https://doi.org/10.1107/S2053229614024218.
[6] C.B. Hübschle, G.M. Sheldrick, B. Dittrich, ShelXle: a Qt graphical user interface for SHELXL, J. Appl. Cryst. 44 (2011) 1281–1284.
[7] D.B. Williams, C.B. Carter, Transmission Electron Microscopy: A Textbook for Materials Science, second ed., Springer Science + Business Media, L.L.C New York, ISBN 978-0-387-76500-6, 2009, pp. 173–193.
[8] P.B. Hirsch, A. Howie, R.B. Nicholson, D.W. Pashley, M.J. Whelan, Electron Microscopy of Thin Crystals, second ed., Robert E. Krieger Publishing Co Inc., Malabar Florida, (1977), pp. 59–61.
[9] R.F. Egerton, P. Li, M. Malac, Radiation damage in the TEM and SEM. Micron 35 (2004) 399–409.
[10] J.R. Fryer, F. Holland, The reduction of radiation damage in the electron microscope, Ultramicroscopy 11 (1983) 67–70.
[11] J.R. Fryer, Radiation damage in organic crystalline films, Ultramicroscopy 14, (1984) 227–236.
[12] J. Strane, L.D. Marks, D.E. Luzzi, M.I. Buckett, J.P. Zhang, B.W. Wessels, Encapsulation, diffusion and DIET in the electron microscope, Ultramicroscopy 25 (1988) 253–258.
[13] S.M. Salih, V.E. Cosslett, Reduction in electron irradiation damage to organic compounds by conducting coatings. Phil. Mag. 30 (1974) 225–228.
[14] A. Howie, The theory of high energy electron diffraction, in: S. Amelinckx, R. Gevers, J. van Landuyt (Eds.), Diffraction and Imaging Techniques in Material Science, Amsterdam: North-Holland, 1978, pp. 457–509.
[15] J.M. Cowley, A.F. Moodie, The scattering of electrons by atoms and crystals. I. A new theoretical approach, Acta Cryst.. 10 (1957) 609–619.
[16] P.A. Stadelmann, EMS - a software package for electron diffraction analysis and HREM image simulation in materials science, Ultramicroscopy 21 (1987) 131–145. JEMS-SaS. Version 4.11531U2022b31 available online: http://www.jems-saas.ch/.
[17] Digital Micrograph Suite 3.4™. Gatan Inc. 5933 Colorado Lane, Pleasanton, CA9458. https://www.gatan.com/products/tem-analysis/gatan-microscopy-suite-software.
